# Case Report: Unilateral biportal endoscopic removal of migrated posterior lumbar interbody cages: a technical note

**DOI:** 10.3389/fsurg.2026.1720261

**Published:** 2026-04-10

**Authors:** Wei Cheng, Dongmei Liu, Hailin Liang, Jiaming Liang, Chengyue Zhu, Rongxue Shao, Dong Wang, Hao Pan, Wei Zhang

**Affiliations:** Department of Orthopedics, Hangzhou Traditional Chinese Medicine Hospital Affiliated to Zhejiang Chinese Medical University, Hangzhou, China

**Keywords:** endoscopic, fusion, migrated interbody cage, revision spine surgery, unilateral biportal endoscopic

## Abstract

**Objective:**

Revision surgery for the removal of a migrated interbody fusion cage is challenging due to scar tissue formation in the surgical area. This study introduces a unilateral biportal endoscopic (UBE) technique for the removal of a migrated fusion cage and reports the patient's clinical outcomes.

**Methods:**

A unilateral lumbar interbody fusion (ULIF) was performed via a trans-facet approach to extract the posterior lumbar interbody cage, thereby bypassing scar tissue from prior surgeries.

**Results:**

The patient's clinical symptoms improved significantly postoperatively, with no complications such as nerve injury or cerebrospinal fluid leakage. At the six-month postoperative follow-up, the patient's lumbar and leg VAS scores showed significant improvement, with no obvious signs of cage loosening observed.

**Conclusion:**

UBE revision surgery may represent a safe and effective alternative for the removal of migrated posterior lumbar interbody cages.

## Highlights

The UBE technique is highly effective for cage removal in revision surgery, as it avoids posterior scar tissue, reduces the risk of intraoperative infection and dural tears, enables removal of displaced cages, and allows placement of expandable, large-size cages under direct endoscopic visualization.

## Introduction

Posterior lumbar interbody fusion (PLIF) is a reliable treatment for lumbar degenerative diseases, with reported rates of cage migration ranging from 2.5% to 6.3% (1). In most such cases, revision surgery is required to remove the migrated cage. Revision surgery is technically demanding due to the presence of scar tissue and previous internal fixation. With advancements in endoscopic technology, the UBE technique has been adopted for lumbar revision surgery due to its clear visualization, flexible instrument manipulation, and minimally invasive nature. Choi et al. (2) described the use of UBE for recurrent lumbar disc herniation and recommended it as an effective approach. Zhu et al. (3) reported favorable outcomes with UBE in revision surgery for adjacent segment disease. Herein, we report the first use of the UBE technique for the removal of a migrated lumbar interbody cage.

## Methods

### Case description

**Figure 1:**
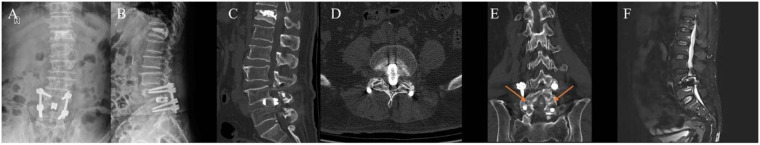
Preoperative imaging showing posterior migration of the L4–5 PLIF cage. **(A)** Anterior/posterior (A/P) radiograph showing the cage centered at L4–5. **(B)** Lateral radiograph showing posterior migration. **(C)** Sagittal CT reconstruction demonstrating posterior migration. **(D)** Axial CT image showing posterior migration. **(E)** Coronal CT scan reveals fusion of the posterolateral articular processes (Red arrow). **(F)** Sagittal MRI showing posterior migration.

A 72-year-old man who had undergone PLIF 13 years earlier developed bilateral lower extremity numbness four years ago following a fall, which resulted in retraction of the titanium cage. Preoperative CT imaging showed no fusion within the intervertebral space. The cage had retracted without evidence of subsidence. Bilateral posterior articular process fusion was observed, with no loosening of the pedicle screws (Figure 1).

### Surgical technique

**Figure 2:**
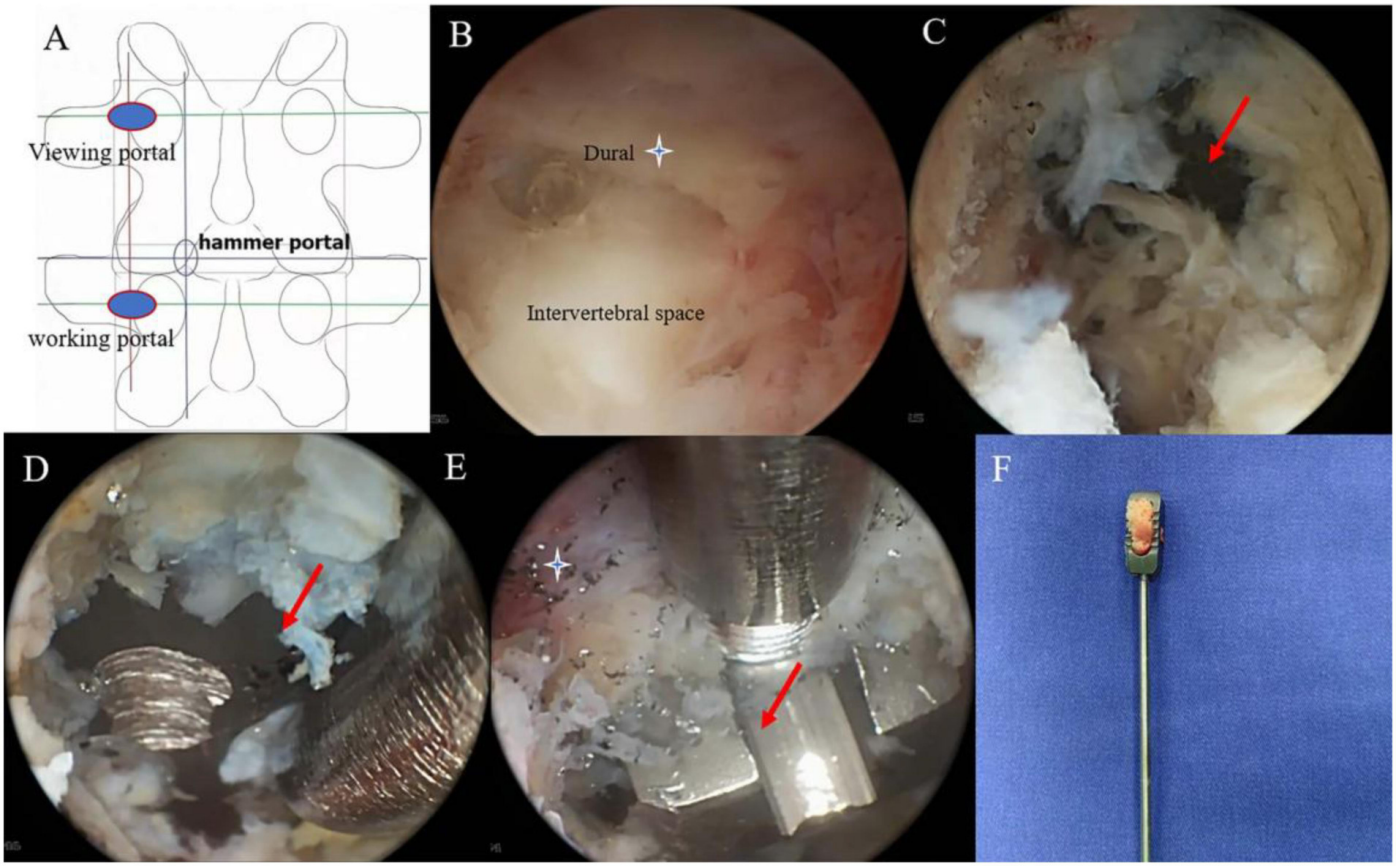
Trans-facet biportal endoscopic approach for removal of the migrated posterior lumbar interbody cage. **(A)** Portal placement schematic. **(B)** Endoscopic view of the dural sac and intervertebral space. **(C)** Lateral view of the migrated cage. **(D)** Intraoperative use of the hammer tool. **(E)** Removal of the migrated cage. **(F)** Removed cage. (The dura mater sac marked with a four-pointed star and the migrated PLIF cage marked with a Red arrow).

The patient was placed in the prone position under general anesthesia. Intraoperative neuromonitoring of somatosensory and motor evoked potentials was performed. Viewing and working portals were established over the pedicles, and a hammer portal is positioned within the safe zone at the level of the intervertebral space, between the lateral border of the facet joint and the medial border of the pedicle. A radiofrequency probe was used to expose the inferior articular process (IAP), which was partially resected to reveal the superior articular process (SAP). The lower portion of the L4 left lamina was removed with a diamond burr until the proximal insertion of the ligamentum flavum was exposed. The ligamentum flavum was carefully separated from scar tissue and resected. Residual nucleus pulposus tissue was visualized, and foraminoplasty was performed progressively using manual reamers (8–12 mm in diameter). Endoscopic visualization of the intervertebral disc revealed the lateral aspect of the migrated cage (Figure 2).

**Figure 3:**
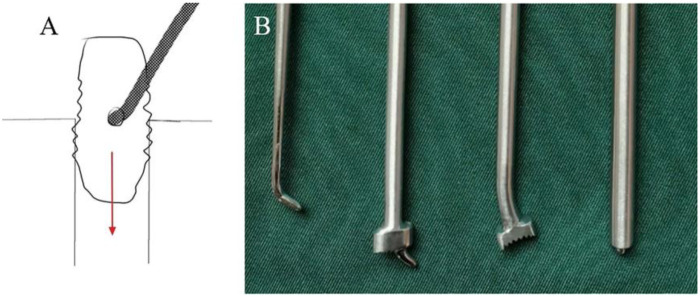
The process of cage loosening. **(A)** Schematic diagram illustrating the use of the hammering tool. **(B)** The tools for loosening the cage.

A cage adjustment tool was introduced through the hammer portal to rotate and advance the cage deeper via hammering, after which a diamond burr was used to grind away any potential callus that may have formed between the cage and the endplates. The tail cap of the cage was exposed, after which the cage was engaged through the working portal under direct endoscopic view. Once securely attached to the handle, the cage was carefully removed (Figure 3).

**Figure 4:**
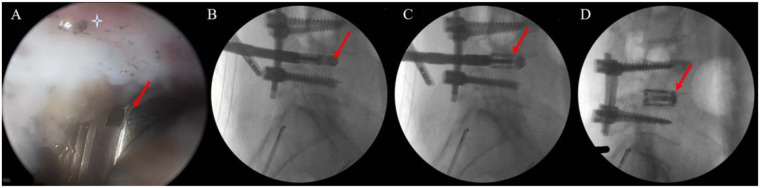
Intraoperative fluoroscopic and endoscopic views during expandable titanium cage insertion. **(A)** Endoscopic view of the cage. **(B–D)** Lateral fluoroscopic images showing the expanded cage positioned at L4–L5. (Cage marked with a red arrow).

Subsequently, a large expandable cage was inserted into the disc space. After confirming correct positioning, the cage was expanded under C-arm fluoroscopic guidance (Figure 4).

## Results

**Figure 5:**
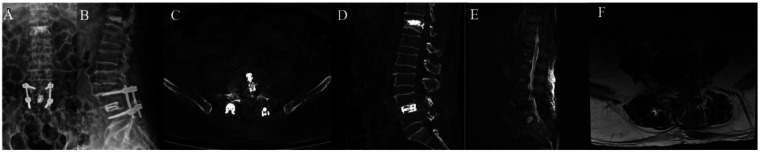
Postoperative imaging. **(A)** A/P and **(B)** lateral radiographs showing the expandable cage at L4–L5. **(C)** Axial and **(D)** sagittal CT images confirming correct cage position. **(E)** Sagittal and **(F)** axial MRI showing no significant spinal cord compression or cerebrospinal fluid leakage.

**Figure 6:**
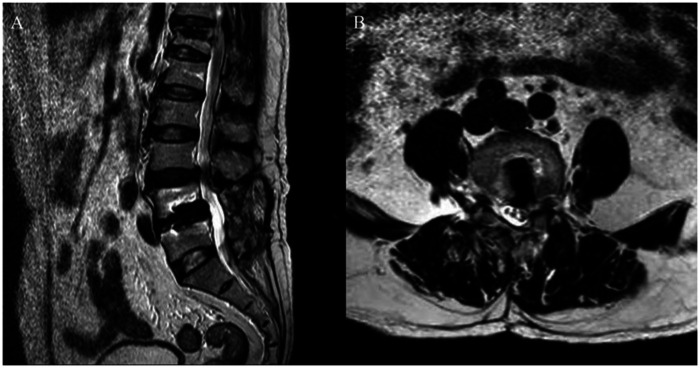
Six-month postoperative MRI showing no significant posterior cage displacement.

Postoperative radiography and CT scans confirmed correct placement of the expandable interbody cage at L4–L5 (Figure 5). The patient's drain was removed 24 hours postoperatively, and he was mobilized on the same day. Follow-up imaging showed no cage loosening or endplate collapse, with improvement in lumbar and lower limb symptoms (Table 1). At the six-month postoperative follow-up, no significant subsidence or retropulsion of the interbody fusion cage was observed in the patient (Figure 6).

**Table 1:** Pre- and postoperative VAS for back and leg scores and ODI scores.

VAS Back/VAS Leg/ODI scores		
Preoperative scores	Postoperative scores at Hospital discharge	Postoperative scores at latest follow-up
6/7/36	3/4/32	2/2/25

## Discussion

Revision surgery for cage migration involves replacing previous graft material, preparing the endplate properly, promoting biological fusion, and improving the biomechanical environment (4). Such procedures are technically demanding. Anterior or anterolateral approaches are often preferred (5, 6), as they facilitate disc space preparation, allow removal of the previous cage, enable insertion of a larger cage, and avoid posterior scar tissue. However, these approaches may be unfeasible if the cage is displaced posteriorly, necessitating alternative techniques.

Previous attempts have been made to use endoscopic spine surgery for revision of migrated lumbar interbody cages. RUDOLF (7) reported successful revision endoscopic spine surgery for three cases with cage migration after lumbar fusion. Two of these cases developed transient radiculitis postoperatively.

In the present case, preoperative CT showed preserved and fully fused facet joints, with the migrated cage located centrally and posteriorly. To restore disc height and prevent anterior column instability, a trans-facet approach was utilized in this procedure, which avoided scar tissue, reduced the risk of nerve injury, and exposed the lateral aspect of the cage, facilitating its rotation and loosening. Despite the advantages of endoscopic visualization, intraoperative neurophysiological monitoring was used due to the high risk of nerve injury. An additional hammer portal was necessary to loosen the migrated cage due to its posterior and off-center position.

When using a posterior approach, the presence of epidural scar tissue makes dural retraction and nerve root decompression challenging. In ULIF, inserting a standard cage through a small incision can be difficult, and using a large cage through the interlaminar space risks neural injury. However, the use of an expandable cage may facilitate safer insertion in ULIF (8). Expandable cages are inserted in a compact form and expanded in situ, reducing the risk of pullout or subsidence while minimizing nerve root injury.

## Limitations

A key limitation of endoscopic revision surgery is that it requires the surgeon to be highly proficient in spinal endoscopy and experienced in open revision procedures. Managing adhered nerves in confined spaces or removing rigid fusion devices demands exceptional skill and adaptability. Not all cases of cage migration are suitable for endoscopic revision; candidates include those with mild posterior displacement, intraspinal retention of the device, or separable nerve adhesions. Contraindications include cases requiring long-segment correction or replacement of long-segment internal fixation.

## Conclusion

In conclusion, UBE may serve as a complementary minimally invasive technique for revision surgery following lumbar interbody fusion.

## Data Availability

The raw data supporting the conclusions of this article will be made available by the authors, without undue reservation.
